# Automated Thrombin Generation Assay for Rivaroxaban, Apixaban, and Edoxaban Measurements

**DOI:** 10.3389/fcvm.2021.717939

**Published:** 2021-09-09

**Authors:** Tamana Meihandoest, Jan-Dirk Studt, Adriana Mendez, Lorenzo Alberio, Pierre Fontana, Walter A. Wuillemin, Adrian Schmidt, Lukas Graf, Bernhard Gerber, Gabriela Monika Maeder, Cédric Bovet, Thomas C. Sauter, Michael Nagler

**Affiliations:** ^1^Department of Epidemiology, Maastricht University, Maastricht, Netherlands; ^2^Department of Clinical Chemistry, Inselspital, Bern University Hospital, and University of Bern, Bern, Switzerland; ^3^Division of Medical Oncology and Hematology, University and University Hospital Zurich, Zurich, Switzerland; ^4^Department of Laboratory Medicine, Cantonal Hospital Aarau, Aarau, Switzerland; ^5^Service and Central Laboratory of Hematology, CHUV, Lausanne University Hospital, Lausanne, Switzerland; ^6^Division of Angiology and Hemostasis, Geneva University Hospital, Geneva, Switzerland; ^7^Division of Hematology and Central Hematology Laboratory, Cantonal Hospital of Lucerne and University of Bern, Bern, Switzerland; ^8^Clinic of Medical Oncology and Hematology and Institute of Laboratory Medicine, City Hospital Waid and Triemli, Zurich, Switzerland; ^9^Centre for Laboratory Medicine St. Gallen, St. Gallen, Switzerland; ^10^Clinic of Hematology, Oncology Institute of Southern Switzerland, Bellinzona, Switzerland; ^11^University of Zurich, Zurich, Switzerland; ^12^Department of Emergency Medicine, Inselspital, Bern University Hospital, Bern, Switzerland; ^13^Department of Hematology, Inselspital, Bern University Hospital, Bern, Switzerland

**Keywords:** diagnostic accuracy, thrombin generation assay, anti-Xa assay, laboratory monitoring, rivaroxaban, apixaban, edoxaban, direct oral anticoagulants

## Abstract

**Background:** The thrombin generation assay (TG) is a promising approach to measure the degree of anticoagulation in patients treated with direct oral anticoagulants (DOAC). A strong association with plasma drug concentrations would be a meaningful argument for the potential use to monitor DOAC.

**Objectives:** We aimed to study the correlation of TG with rivaroxaban, apixaban, and edoxaban drug concentrations in a large, prospective multicenter cross-sectional study.

**Methods:** Five-hundred and fifty-nine patients were included in nine tertiary hospitals. The Technothrombin® TG was conducted in addition to an anti-Xa assay; LC-MS/MS was performed as the reference standard.

**Results:** Correlation (r_s_) between thrombin generation measurements and drug concentrations was −0.72 for peak thrombin generation (95% confidence interval, CI, −0.77, −0.66), −0.55 for area under the curve (AUC; 95% CI −0.61, −0.48), and 0.80 for lag time (95% CI 0.75, 0.84). In contrast, r_s_ was 0.96 with results of the anti-Xa activity (95% CI 0.95–0.97). Sensitivity with regard to the clinically relevant cut-off value of 50 μgL^−1^ was 49% in case of peak thrombin generation (95% CI, 44, 55), 29% in case of AUC (95% CI, 24, 34), and 64% in case of lag time (95% CI, 58, 69). Sensitivity of the anti-Xa assay was 95% (95% CI, 92, 97).

**Conclusions:** The correlation of thrombin generation measurements with DOAC drug concentrations was weak, and clinically relevant drug levels were not predicted correctly. Our results do not support an application of TG in the monitoring of DOAC.

**Graphical Abstract:**
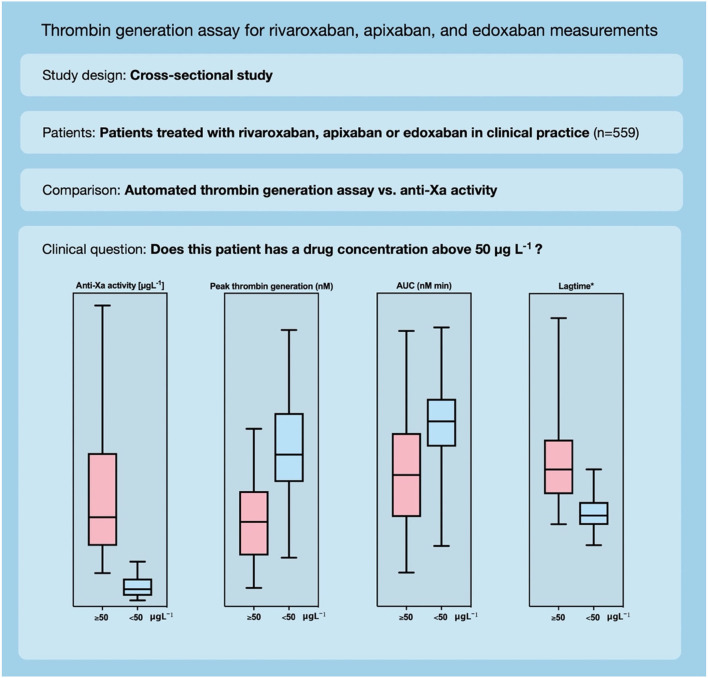
Automated thrombin generation assay for rivaroxaban, apixaban, and edoxaban measurements.

## Introduction

Direct oral anticoagulants (DOAC), including the anti-Xa inhibitors rivaroxaban, apixaban, and edoxaban, have simplified and improved health care in many patients worldwide (1). However, whether or not drug monitoring would improve patient outcomes remains unclear. Vitamin K antagonists (VKA) and unfractionated heparin have been successfully applied for decades, and treatment without monitoring would not have been conceivable (2). Low molecular weight heparins (LMWH), which share some similarities in the mechanism of action with anti-Xa inhibitors, are applied using weight-adapted dosages without the need for regular monitoring (3). Indeed, various arguments for the monitoring of DOAC appear (4). First, an association between drug exposure and bleeding events was observed in some studies (5) and between trough concentrations and thromboembolic events such as strokes in other studies (6, 7). Second, several clinical factors (e.g., renal failure, liver failure, age > 75 years) and several drug interactions (e.g., P-glycoprotein and CYP3A4-modulating substances) significantly affect drug concentrations (8–12). Third, a number of observational studies observed a relevant inter-individual variation of the drug concentration (13–15). Other authors even summarized previous data to suggest an optimal risk-benefit range pointing to a potential target range based on drug concentrations (16). However, the optimal laboratory assay to be used for monitoring of DOAC is to be determined.

Different laboratory tests are being used to measure DOAC drug concentrations in special clinical situations such as major bleeding, urgent surgery, or suspected accumulation (17, 18). The anti-Xa inhibitors rivaroxaban, apixaban, and edoxaban can be determined using chromogenic anti-Xa assays (19). These tests, which are successfully used to determine unfractionated and low molecular weight heparins, were further developed and successfully studied in various evaluation studies (15, 20). Routine coagulation tests such as prothrombin time are not sensitive to detect clinically relevant concentrations of anti-Xa inhibitors (21). The thrombin inhibitor dabigatran can be captured using the thrombin time, an extremely sensitive routine coagulation test available in many institutions (18). Diluted thrombin times have been developed to measure dabigatran drug concentrations (22, 23). Besides, the ecarin clotting time can be utilized to estimate dabigatran drug concentrations (19, 22).

The thrombin generation assay (TG) is regarded as a promising approach for monitoring DOAC anticoagulant activity (24). It can record the amount and kinetics of thrombin generated at a particular point in time (25). Thrombin is the key enzyme of the coagulation cascade, which is generated following factor X activation. The generation of thrombin controls, among other functions, the amount of fibrin generated and thus triggers a stable clot (26). The TG measures the cleavage of a fluorogenic substrate over time, resulting in a characteristic curve describing the thrombin generation activity. The parameters of the TG are retrieved from this curve: the maximum concentration of thrombin (*thrombin peak height*), the total amount of thrombin generation *(area under the curve; AUC)*, and the time to initiation of the exponential phase *(lag time)*. Following these considerations, one might hypothesize that TG is an elegant, functional assay to be used to monitor DOAC, including the anti-Xa inhibitors rivaroxaban, apixaban, and edoxaban. A strong correlation of TG measures with anticoagulant drug concentrations would be a strong argument for a potential application in monitoring DOAC.

### Aims

We aimed to study the association of state-of-the-art thrombin generation measurements with rivaroxaban, apixaban, and edoxaban drug concentrations, and compare the results with the performance of an anti-Xa assay in a prospective multicenter cross-sectional study conducted in routine clinical practice.

## Methods

### Design, Setting, and Population

**Figure 1:**
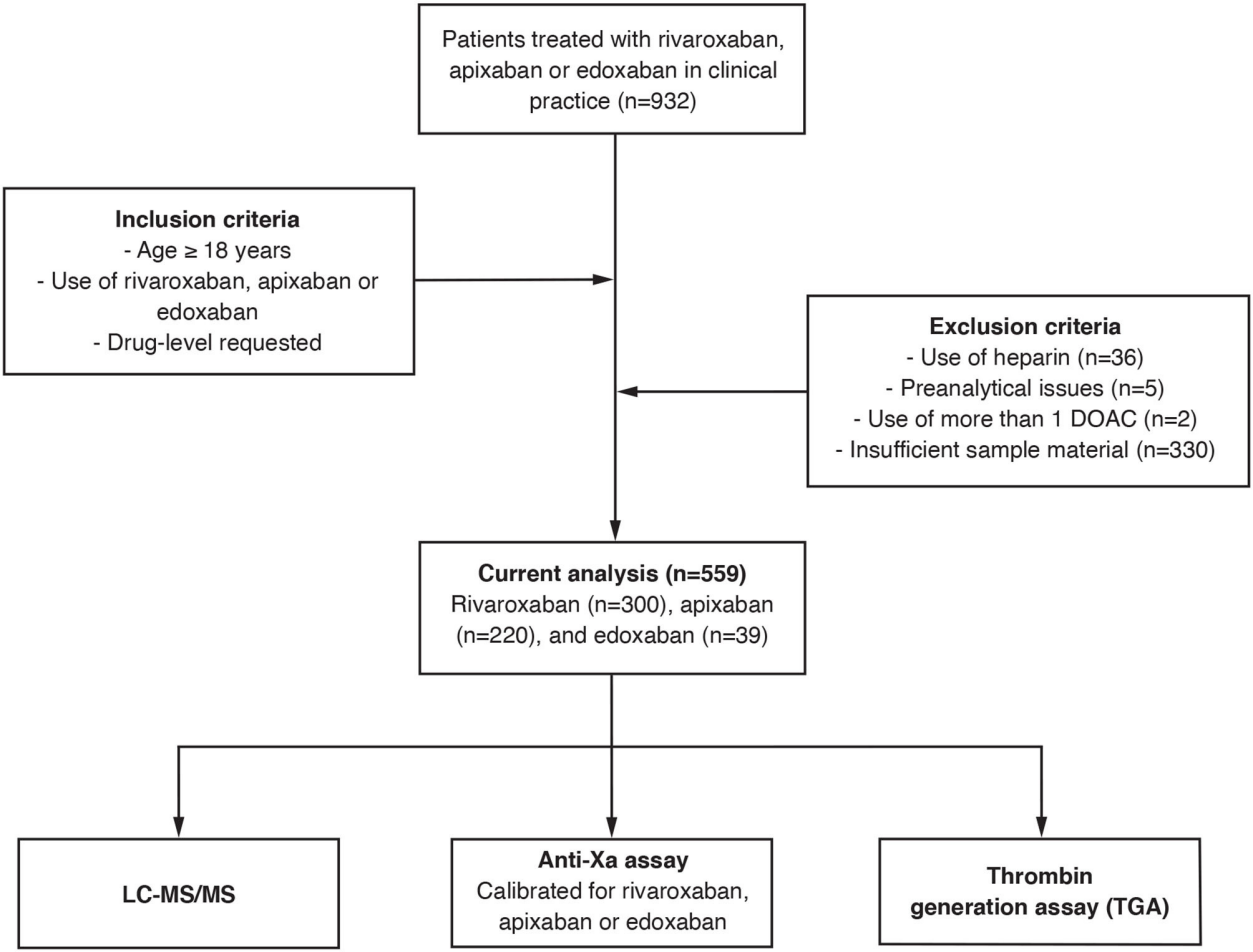
Flow of the patients. A prospective, multicentre cross-sectional study was conducted to study the association of a state-of-the-art thrombin generation assay with rivaroxaban, apixaban, and edoxaban drug concentrations in contrast to the association obtained with an anti-Xa assay.

This is a multicenter cross-sectional study conducted between 2018 and 2019 (20). Patients treated with rivaroxaban, apixaban, or edoxaban in routine clinical practice were included in nine specialized hemostasis laboratories affiliated to tertiary hospitals in Switzerland. Inclusion criteria were (a) age above 18 years, (b) use of rivaroxaban, apixaban, and edoxaban, (c) DOAC drug level requested by the treating physician, and (d) signed general informed consent, if requested by the local authorities. Exclusion criteria were (a) refused general informed consent, (b) use of heparin, (c) preanalytical issues, (d) intake of more than one DOAC, and (e) insufficient sample material. To cover the full range of drug levels observed in clinical practice, we collected samples regardless of the time of last drug intake. The study design is illustrated in Figure 1 (CONSORT flow diagram). Ultra-performance liquid chromatography-tandem mass spectrometry (LC-MS/MS) was used as reference (gold) standard because it is regarded as the most accurate technique to measure DOAC drug concentrations (27). The study was approved by the appropriate ethical committees and all participating institutions. The study was conducted in accordance with the declaration of Helsinki.

### Data Collection and Handling of Samples

Protocols were implemented at all participating institutions to ensure adequate pre-analytic conditions (28). Blood samples were drawn in plastic tubes containing 1 mL trisodium citrate (0.106 mol L^−1^) per mL of blood. Samples were centrifuged using an established scheme (28), and aliquots were immediately snap-frozen at −20°C and stored at −80°C until shipment. Samples were sent on dry ice to the central laboratory and delivered within 3–4 h. Samples were kept frozen until the determination of laboratory tests without any freeze-thaw cycles. All laboratory test results were exported automatically to avoid any typing errors. Coded clinical and laboratory data were stored in a secured RedCAP database. The coding list was stored separately with access by the principal investigator only. The following data were collected: age, sex, and drug.

### Thrombin Generation Measurements

An automated thrombin generation assay with high precision characteristics was selected for this study. The Ceveron® TGA RC High (Technoclone, Vienna, Austria) was used on a Ceveron® t100 analyzer (Technoclone, Vienna, Austria), and the manufacturer's instructions were strictly followed. The responsiveness of the Ceveron® TGA RC High trigger reagent for the measurement of direct oral anticoagulants was confirmed in a previous study using spiked samples (29). All lyophilized reagents and calibrators were brought to room temperature, reconstituted with distilled water, and gently mixed. A 4-point calibration curve was generated using the Ceveron® TGA CAL set (calibrated against the Thrombin Reference Preparation of the WHO). Before each test run, internal quality control was done using the Ceveron® TGA CONT H and L vials (lyophilized human plasma with increased or decreased thrombin generation). Between-run impression was determined by 19 runs with commercially available frozen normal human plasma (Technoclone, Vienna, Austria). The samples were thawed for 15 min in a water bath at 37°C and analyzed immediately. To patient's plasma (40 μL), Ceveron® TGA BUF (Tris-Hepes-NaCl buffer), Ceveron® TGA RC High trigger reagent (high concentration of phospholipid micelles containing recombinant human tissue factor in Tris-Hepes-NaCl buffer), Ceveron® TGA SUB (fluorogenic substrate 1 mM Z-G-G-R-AMC), and 25 mM CaCl2 were added to give 150 μL final reaction mixture. Even though precise concentrations of tissue factor and phospholipids are not given, tissue factor ranges between 20 and 50 pM and phospholipids around 5 μM. The fluorescent substrate was monitored by the Ceveron® t100 TG software, calculating thrombin generation over time. The following parameters were used as readouts: (a) peak thrombin generation (nmol); (b) the lag time (min); and (c) the area under the curve (AUC; nmol^*^min). Frozen normal human plasma was analyzed in line with patients' samples in each test run.

### Determination of the Anti-Xa Activity

The TECHNOCHROM® anti-Xa assay was used for determining the anti-Xa activity, using rivaroxaban, apixaban, and edoxaban calibrators (Technoclone, Vienna, Austria). Measurements were done on a Ceveron® t100 analyzer (Technoclone, Vienna, Austria). Samples were thawed for 15 min in a water bath at 37°C and analyzed immediately. For DOAC concentrations ≤ 150 ng/mL, samples, calibrators, and controls were assayed in a 1:5 dilution and for concentrations ≥150 ng/mL in a 1:20 dilution. The instructions of the manufacturer were strictly followed. The kit consists of 20 mL reagent 1 (anti-Xa buffer, TRIS-EDTA buffer, pH 8.4), 4 mL reagent 2 (Bovine Factor Xa), and 4 mL reagent 3 (Chromogenic substrate). All reagents had reached room temperature before use, bovine factor Xa, and the substrate was preheated to 37°C. Reagent 2 and 3 were separately reconstituted in 4 mL distilled water. The following substances were incubated in a well of a microtiter plate, incubated at 37°C: 200 μL of the diluted sample, 200 μL of bovine factor Xa (mixed and incubated at 37°C for 60 s), 200 μL of Xa substrate (mixed and incubated at 37°C for 30 s), and 200 μL 2% citric acid (mixed and measured absorbance at 405 nm).

### Determination of the LC-MS/MS

Rivaroxaban, apixaban, edoxaban, and edoxaban M4 were quantified by LC-MS/MS as previously described (20). Briefly, protein precipitation and analyte extraction were performed by adding to the plasma acetonitrile:water 1:1 (v/v), extraction buffer (MassTox TDM Series A, Chromsystems, Gräfelfing, Germany), and precipitation reagent (MassTox TDM Series, Chromsystems, Gräfelfing, Germany) containing the isotope labeled internal standards (13C6 rivaroxaban, 13CD3 apixaban, 13CD2 edoxaban) The samples were vortexed and then centrifuged at 14000 rcf and 20°C for 4 min. The supernatant was diluted with water: methanol 8:2 (v/v) and stored at 10°C until analysis. Calibrators and QCs were prepared in pooled plasma. Then, the extracted samples were analyzed by reversed-phase chromatography on a triple quadrupole mass spectrometer (Xevo TQ-S, Waters, Milford, USA) coupled to a UPLC Acquity I-Class system (Waters, Milford, USA). Edoxaban M4 concentration was summed up with edoxaban concentration for further analysis.

### Statistical Analysis

The demographic characteristics were described using proportions and percentages and a median and interquartile range (IQR) for the continuous variables. The accuracy of the TG assay and the anti-Xa assay was determined by calculating Spearman's correlation coefficient in relation to the plasma concentration as measured by LC-MS/MS (overall and per drug). A correlation coefficient of r_s_≥0.9 was considered as accurate (alternative hypothesis). The Deming regression was used to describe the linear relationship, and a Bland-Altman plot was created to observe a potential bias over the spectrum of measurements (done for the anti-Xa activity only) (30). The systematic differences between the assay are analyzed by calculating the mean difference and the SD to compute 95% limits of agreement for every level of measurements (average difference ± 1.96 standard deviation of the difference) (31). To assess the diagnostic accuracy of the TG and the anti-Xa assay in terms of clinical utility, we determined their sensitivity and specificity with regard to the clinically relevant drug level 50 μg/L, used as a cut-off below which most invasive procedures allowed. A sensitivity of at least 90% and a specificity of more than 80% were regarded as adequate. All statistical analyses were performed using RStudio (1.3. 1093-1); figures were created using Prism 8 (GraphPad Software, Inc., La Jolla, CA, USA).

## Results

### Patient Characteristics

Nine-hundred and thirty-two patients were included in this prospective multicenter cross-sectional study; the CONSORT flow diagram is given in Figure 1. From this population, 36 patients were excluded because of heparin use, five patients due to pre-analytical issues, two patients because of using more than one DOAC, and 330 patients due to insufficient sample material. Eventually, samples of 559 patients were used for the current analysis. Of those, 300 patients used rivaroxaban, 220 apixaban, and 39 edoxaban. The median age was 75 years (IQR, 65–82 years) and 41.5% of the patients were female. Details are given in Table 1.

**Table 1:** Baseline characteristics of patients treated with rivaroxaban, apixaban or edoxaban (*n* = 559).

	**Patients treated with**				
	**Rivaroxaban**	**Apixaban**	**Edoxaban**	**All**	**Missing data**
Patients (n/%)	300 (53.7)	220 (39.4)	39 (7.0)	559 (100)	0
Age (median/IQR)	73 (62–82)	78 (67–82)	75 (63–80)	75 (65–82)	91
Sex (n/%)					7
Male	173 (57.9)	128 (59.5)	22 (59.5)	323 (58.5)	–
Female	126 (42.1)	87 (40.5)	16 (40.5)	229 (41.5)	–

*N, number; IQR, interquartile range*.

### Association Between Thrombin Generation Measurements and Drug Concentrations

**Figure 2:**
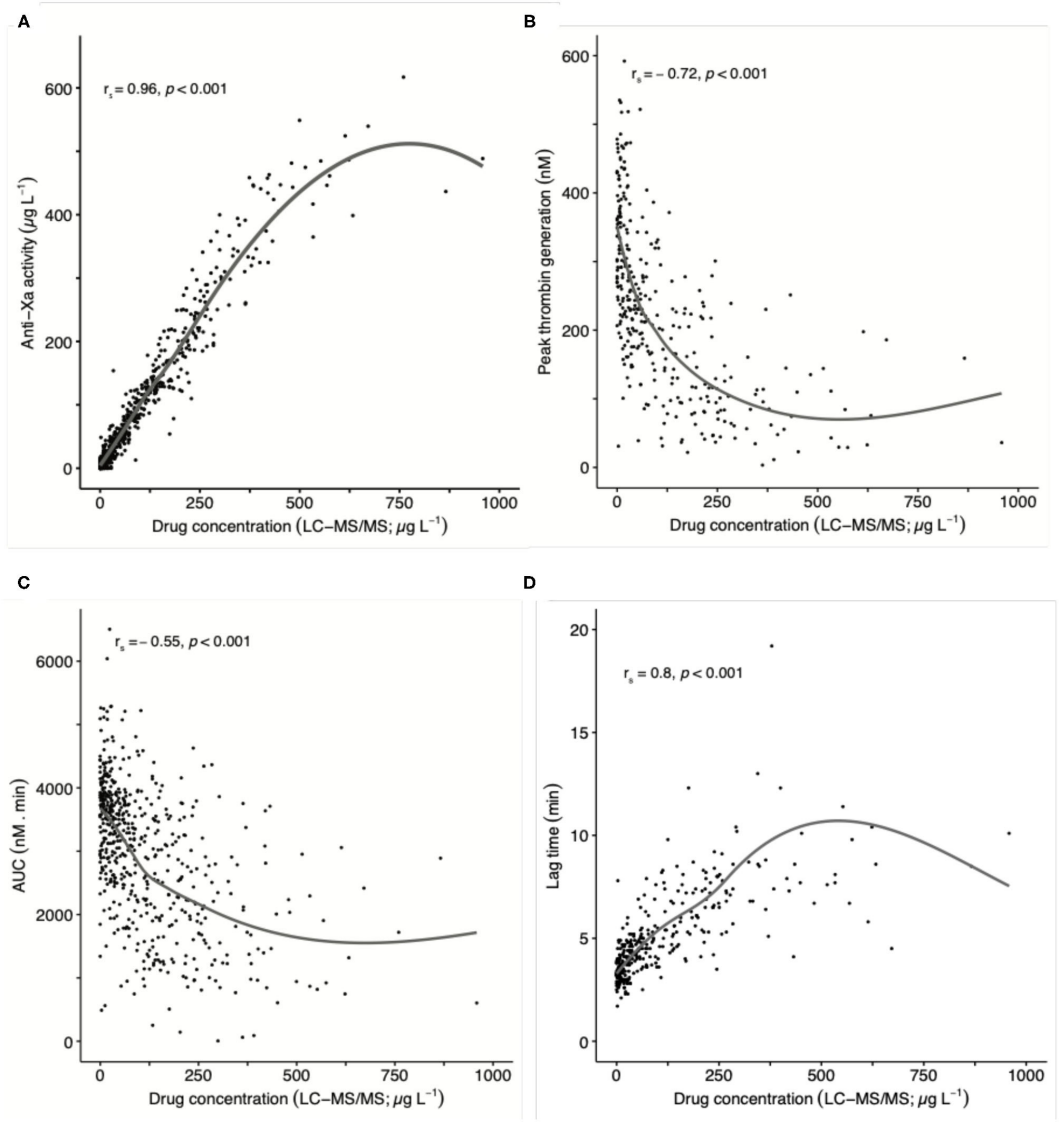
Association of thrombin generation results and anti-Xa measurements with drug concentration in 559 patients taking rivaroxaban, apixaban, or edoxaban in clinical practice (pooled data): **(A)** anti-Xa activity, **(B)** peak thrombin generation, **(C)** area under the curve (AUC), and **(D)** lag time. Ultra-high performance liquid chromatography-tandem mass spectrometry (LC-MS/MS) was used to determine drug levels. The overall Spearman's correlation coefficient (r_s_) was 0.96 in case of anti-Xa measurements, −0.72 in case of peak thrombin generation, −0.55 in case of AUC, and 0.80 in case of lag time. A non-linear (smooth) curve is given to illustrate the association.

The association between thrombin generation measurements and drug concentrations are illustrated in Figures 2B–D. The correlation coefficient (r_s_) was −0.72 for peak thrombin generation (95% confidence interval, CI, −0.77, −0.66), −0.55 for area under the curve (AUC; 95% CI −0.61, −0.48), and 0.80 for lag time (0.75, 0.84). The slope of the regression line was −0.72 for peak thrombin generation (95% CI −0.95, −0.56), −15.89 for AUC (−19.04, −13.57), and 0.01 for lag time (0.01, 0.02), see Table 2. The Y-intercept was 313.9 for peak thrombin generation (95% CI 292.5, 339.1), 4842.3 for AUC (4607.2, 5154.9), and 3.9 for lag time (3.6, 4.1). Supplementary Table 1 of the Supplemental Material reports the results for the individual drugs. Determining the between-run imprecision (19 runs), the coefficient of variation (CV) was 7.4% for peak thrombin generation, 4.2% for AUC, and 7.1 % for lag time.

**Table 2:** Accuracy of anti-Xa measurements and thrombin generation results with regard to drug concentration in 559 patients taking rivaroxaban, apixaban, or edoxaban in clinical practice.

**Measurement**	**Anti-Xa activity (μg L^**−1**^)**	**Peak thrombin generation (nM)**	**Area under the curve (nM × min)**	**Lag time (min)**
Spearman's correlation coefficient (95% CI)	0.96 (0.95, 0.97)	−0.72 (−0.77, −0.66)	−0.55 (−0.61, −0.48)	0.80 (0.75, 0.84)
Deming regression slope (95% CI)	0.81 (0.73, 0.88)	−0.72 (−0.95, −0.56)	−15.89 (−19.04, −13.57)	0.01 (0.01, 0.02)
Y-intercept (95% CI)	13.8 (7.1, 20.4)	313.9 (292.5, 339.1)	4842.3 (4607.2, 5154.9)	3.9 (3.6, 4.1)
Bland-Altman difference plotBias (95% CI)	8.93 (3.71, 14.15)	NA	NA	NA
Lower limit of agreement (95% CI)	−86.86 (−95.90, −77.82)	NA	NA	NA
Upper limit of agreement (95% CI)	104.72 (95.68, 113.76)	NA	NA	NA

*Ultra-performance liquid chromatography-tandem mass spectrometry (LC-MS/MS) was used to determine drug levels. The Spearman's correlation coefficient is given (r_s_), coefficients of Deming regression, as well as measures of the Bland-Altman difference plot*.

### Association Between Anti-Xa Activity and Drug Concentrations

Figure 2A illustrates the association between anti-Xa activity and drug concentrations. The correlation coefficient (r_s_) was 0.96 (95% CI 0.95–0.97). The slope of the regression line was 0.81 (95% CI 0.73, 0.88) and the Y-intercept was 13.8 (7.1, 20.4), see Table 2. The bias of the Bland-Altman difference plot is 8.9 (95% CI 3.7, 14.2) with a lower limit of agreement of −86.9 (−95.9, −77.8) and upper limit of agreement of 104.7 (95.7, 113.8). See Supplementary Figure 1 and Supplementary Table 1.

### Diagnostic Accuracy Regarding Clinically Significant Drug Levels

**Figure 3:**
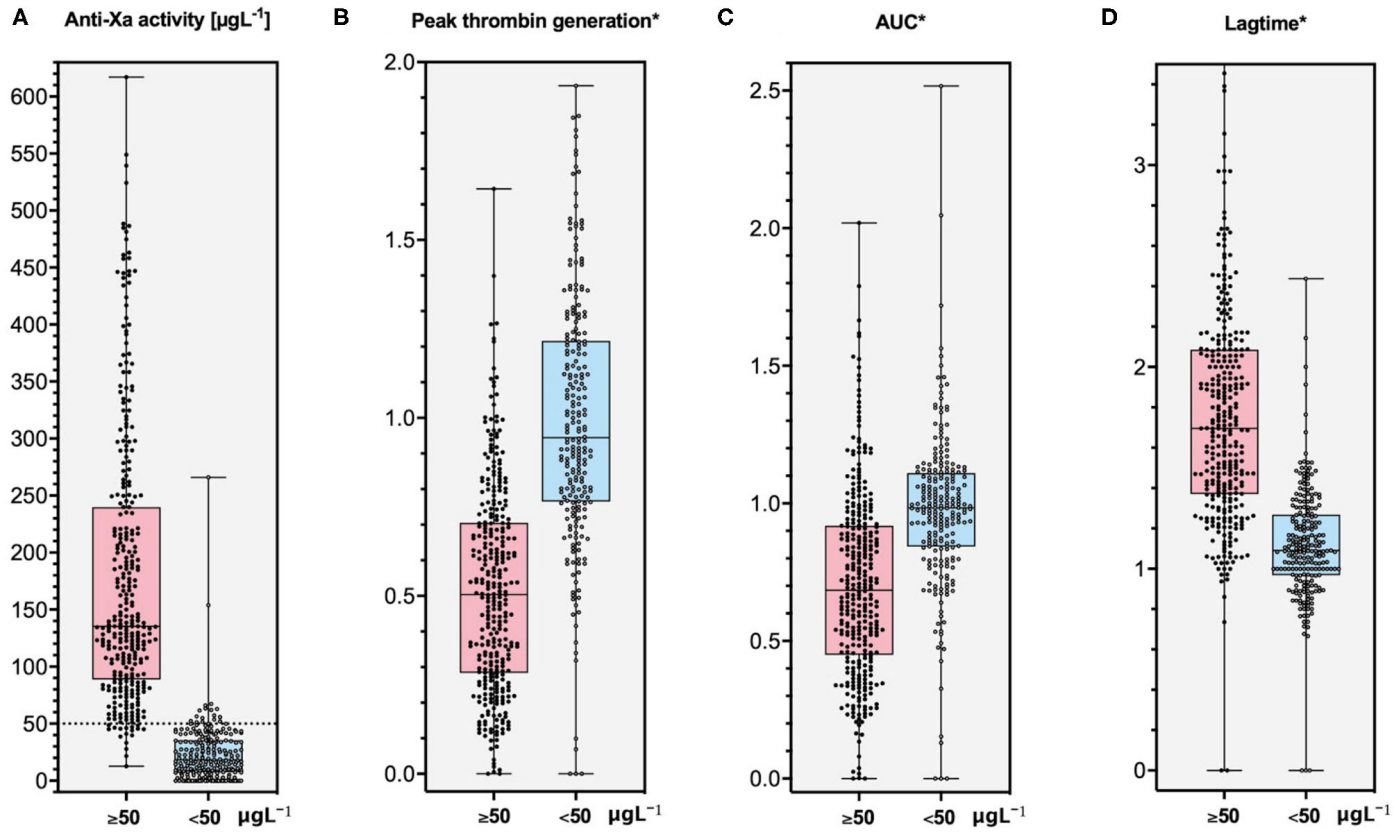
Distribution of thrombin generation results and anti-Xa measurements in patients with and without clinically relevant concentrations of rivaroxaban, apixaban, and edoxaban (50 μg L^−1^): **(A)** anti-Xa activity, **(B)** peak thrombin generation, **(C)** area under the curve (AUC), and **(D)** lag time. *Thrombin generation results are expressed in relation to measurements obtained with standardized human plasma (for example: AUC patients/AUC SHP). Ultra-high performance liquid chromatography-tandem mass spectrometry (LC-MS/MS) was used to determine drug levels. The sensitivity was 83% (95% CI, 78, 87) in case of peak thrombin generation (cut-off level was 80% of normal plasma), 61% (56, 66) in case of AUC (80% of normal plasma), 89% (85, 92) in case of lag time (120% of normal plasma), and 95% in case of anti-Xa activity (95% CI, 92, 97). The specificity was 70% (95% CI, 64, 76) in case of peak thrombin generation, 78% (72, 83) in case of AUC, 66% (60, 72) in case of lag time, and 92% in case of anti-Xa activity (95% CI, 88, 95).

**Figure 4:**
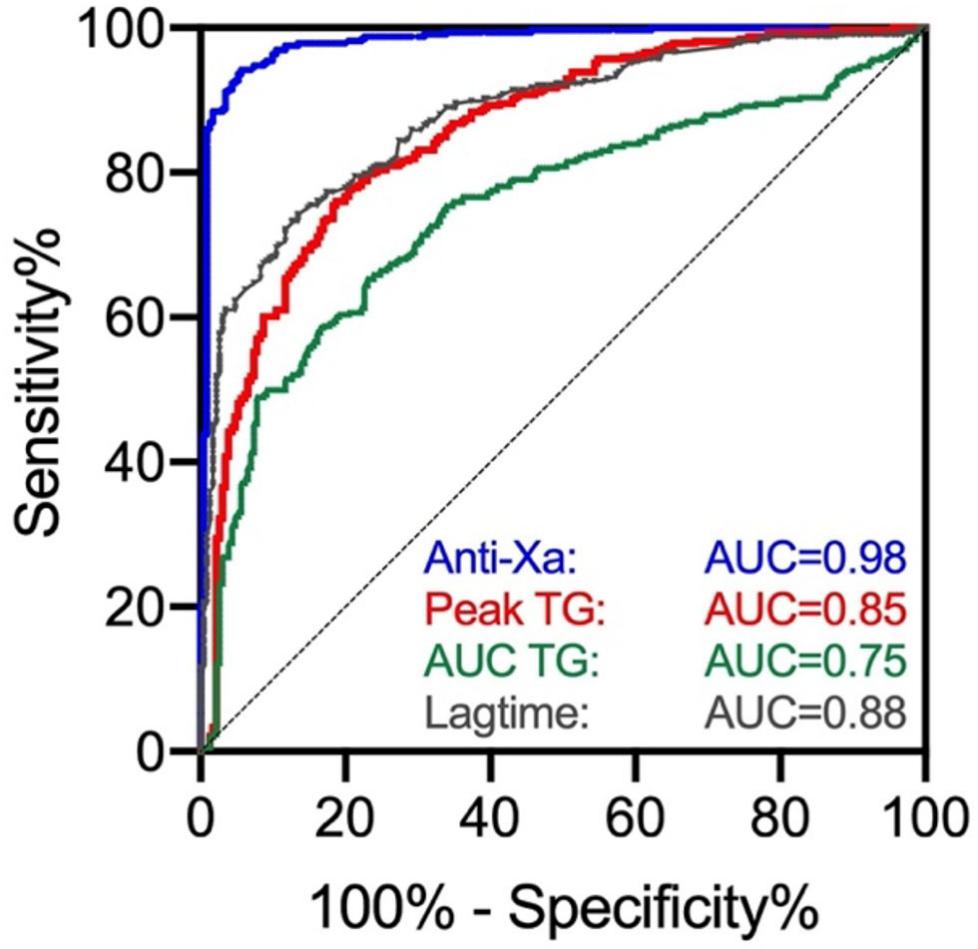
Receiver operating characteristics (ROC) curve of thrombin generation results and anti-Xa measurements for clinically relevant concentrations of rivaroxaban, apixaban, and edoxaban (50 μg L^−1^). AUC was 0.98 for anti-Xa measurements, 0.85 for peak thrombin generation, 0.75 for AUC, and 0.88 for lag time.

The distribution of measurements in patients with and without clinically relevant drug levels (50 μgL^−1^) is shown in Figure 3. Using 80% of peak thrombin generation measured in standardized normal plasma as a cut-off, the sensitivity was 83% (95% CI, 78, 87), and the specificity was 70% (64, 76). AUC measurements resulted in a sensitivity of 61% (95% CI, 56, 66) and a specificity of 78% (72, 83). Sensitivity of lag time measurements at a cut-off of 120% of standardized normal plasma was 89% (95% CI, 85, 92), and the specificity was 66% (60, 72). In contrast, the sensitivity was 95% in case of anti-Xa measurements (95% CI, 92, 97) and specificity was 92% (88, 95), respectively. Receiver-operating characteristics are given in Figure 4.

As a sensitivity analysis, we used 50% of standardized normal plasma as an additional cut-off. In this case, the sensitivity of peak thrombin generation measurements was 49% (95% CI, 44, 55) and the specificity was 93% (90, 96). The sensitivity of AUC measurements was 29% (95% CI, 24, 34) and the specificity 96% (92, 98). For lag time measurements, the sensitivity was 64% (95% CI, 58, 69) as a cut-off of 150% the standardized normal plasma, and the specificity was 94% (90, 96).

## Discussion

This study reports 559 patients treated with rivaroxaban, apixaban, or edoxaban in routine clinical practice, which were studied in a cross-sectional study analyzing thrombin generation assay and anti-Xa activity in comparison to LC-MS/MS. The correlation of thrombin generation measurements with drug concentrations was weak, and clinically relevant drug levels were not predicted correctly. In contrast, the correlation of anti-Xa measurements with drug concentrations was high, and relevant drug levels were predicted correctly.

Thrombin generation assay was conducted in the presence of DOAC in a number of studies. Various *in-vitro* or *ex-vivo* studies using spiked samples demonstrated that the presence of DOAC changed TG measures (32–36). In particular, Khoo et al. observed TG measures in 8 patient samples spiked with dabigatran before and after adding activated prothromplex concentrate (37). Bloemen et al. performed *in-vitro* and *ex-vivo* measurements in 63 patients and studied the effect of dabigatran in the presence and absence of idarucizumab (38). Schenk et al. observed in 20 *in-vitro* and *in-vivo* patient samples that rivaroxaban was significantly correlated with thrombin generation parameters (39). Tripodi et al. observed in 20 samples from healthy volunteers that apixaban affects all thrombin generation parameters (40). Sinegre et al. observed a change in TG parameters in 87 adult and 97 children patient samples spiked *in-vitro* with edoxaban (41). Samama et al. demonstrated concentration-dependent effects of edoxaban in spiked patient samples (42). Similar results were also observed in a randomized, placebo-controlled cross-over study including patients taking rivaroxaban (43). Pfrepper et al. included 380 samples from patients taking apixaban, dabigatran, edoxaban, or rivaroxaban and correlated anti-Xa measurements with TG parameters (44). Similar to these studies, we observed changes in TG measures in patients treated with rivaroxaban, apixaban, and edoxaban. However, correlation of TG parameters with drug concentrations was weak to moderate only.

In contrast to previous studies, we included a large number of patients taking rivaroxaban, apixaban, or edoxaban in clinical practice. LC-MS/MS was conducted as a reference standard in all patient samples. Besides, the sensitivity and specificity of TG measurements were calculated against established cut-offs levels. Anti-Xa activity was measured in parallel to compare the results with an assay used routinely in most tertiary hospitals. In addition, patients were recruited from nine tertiary hospitals in Switzerland, which increases the external validity and makes the samples more representative for the general patient population. Therefore, selection bias is considered to be unlikely (45). However, several limitations must be noted. First and most important, we did not record clinical outcomes in terms of bleedings and thromboembolic events. As long as these events are rare, a study like this would need a much larger patient population and a very long observation period. Second, the number of patients is limited in certain subgroups such as lower concentrations of edoxaban. Even though there is no indication that the effects observed might be different in these subgroups, we cannot fully exclude such an effect. Third, our results were obtained with one specific TG device and reagent and the results might be different with other analyzers. However, this is a device of the latest generation with a high precision and there is no hint that this assay could perform worse than others. Fourth, thrombin generation measurements were conducted using frozen citrated samples, and we cannot entirely exclude that the results might be different in whole blood or fresh samples. However, all pre-analytical requirements were strictly addressed, and we do not believe that this might have entirely changed the results.

Following our data, we have to reject the hypothesis that TG measurements adequately reflect DOAC drug concentrations. The association of thrombin generation measurements with drug concentrations was weak, and clinically relevant drug levels were not predicted correctly. We consider our data to be robust because they were obtained in the most accurate study conducted so far. Therefore, our results represent a clear argument against a potential application of TG in monitoring DOAC. One might argue that TG unfolds its utility for monitoring DOAC by assessing the biological efficacy rather than the drug concentration. Several central arguments can be raised supporting this proposition: First, TG reflects the mechanism of thrombus formation more than any other laboratory test (discussed above). Secondly, TG measurements vary among patients with similar drug levels, potentially reflecting different degrees of baseline pro-thrombotic tendency (4). Thirdly, anticoagulant treatment at a given dosage is not effective in some clinical settings representing high-risk pro-thrombotic situations [e.g., DOAC in patients with antiphospholipid antibody syndrome (46); vitamin K-antagonists in cancer patients (47)]. However, this claim must be supported with data demonstrating a relevant association between TG measurements and clinical outcomes in terms of thromboembolic or bleeding events in anticoagulated patients. To the best of our knowledge, these data are not (yet) available. In contrast, the correlation between anti-Xa activity and drug concentrations was strong in our study as well as in others, and an association between anti-Xa activity and clinical outcomes have been shown in various clinical settings (5–7, 48–50).

## Conclusion

We report results of a large prospective study including patients treated with rivaroxaban, apixaban, or edoxaban in routine clinical practice. The correlation of thrombin generation measurements with DOAC drug concentrations was weak, and clinically relevant drug levels were not predicted correctly. Our results do not support an application of TG in the monitoring of DOAC.

## Data Availability Statement

The raw data supporting the conclusions of this article will be made available by the authors, without undue reservation.

## Ethics Statement

The studies involving human participants were reviewed and approved by Kantonale Ethikkommission Bern. The patients/participants provided their written general informed consent to participate in this study.

## Author Contributions

TM analyzed the data and wrote the manuscript. J-DS, AM, LA, PF, WW, AS, LG, BG, CB, GM, and TS collected data, contributed to study design, protocol, and preparation of the manuscript. GM conducted the LC-MS/MS measurements. MN designed the study, wrote the protocol, collected and analyzed the data, and wrote the manuscript. All authors contributed to the article and approved the submitted version.

## Funding

The study was supported by a research Grant of the Research Fund Hematology Cantonal Hospital Lucerne. MN was supported by a research grant of the Swiss National Science Foundation (#179334). Implementation of the LC-MS/MS measurements was supported by the Gottfried & Julia Bangerter-Rhyner Stiftung (applicant Ursula Amstutz).

## Conflict of Interest

We thank the following companies for the provision of reagents and/or pure substances: Bayer Healthcare AG, Bristol-Myers Squibb, and Daiichi Sankyo, Technoclone. These companies had no role in study design, data collection and analysis, the decision to publish, or manuscript preparation. MN reports research grants from Bayer Healthcare, outside of the submitted work, lecture honoraria from Bayer Healthcare, and Daiichi Sankyo. LA reports research grants from Bayer, CSL-Behring, Novartis, Novo Nordisk, Roche, Sobi, and Takeda. WW reports research grants from Bayer Healthcare, BMS-Pfizer, Daiichi Sankyo and Sanofi, and honoraria for participating in scientific advisory boards from Bayer, Pfizer, and from Alexion Pharma GmbH, all outside the submitted. J-DS reports lecture fees and advisory honoraria from Bayer Healthcare, Pfizer, Takeda, Siemens, and Sanofi. The study was supported by Technoclone, Vienna, Austria with reagents, analyzers, and technical expertise. The remaining authors declare that the research was conducted in the absence of any commercial or financial relationships that could be construed as a potential conflict of interest.

## Publisher's Note

All claims expressed in this article are solely those of the authors and do not necessarily represent those of their affiliated organizations, or those of the publisher, the editors and the reviewers. Any product that may be evaluated in this article, or claim that may be made by its manufacturer, is not guaranteed or endorsed by the publisher.
