# Supplementary material for: Automated Thrombin Generation Assay for Rivaroxaban, Apixaban, and Edoxaban Measurements
Source: Front Cardiovasc Med. 2021 Sep 9;8:717939. doi: 10.3389/fcvm.2021.717939 (PMC8459937; doi:10.3389/fcvm.2021.717939)
Supplement: Supplementary file 1 [file Data_Sheet_1.PDF]

Supplementary material.

T. Meihandoest et. al.

Automated thrombin generation assay for rivaroxaban, apixaban, and edoxaban measurements

Table S1. Accuracy of anti-Xa measurements and thrombin generation results regarding drug concentration in patients taking rivaroxaban, apixaban, or edoxaban: Sensitivity analysis according to drug.

	Anti-Xa activity (μg L^{-1})	Peak thrombin generation (nM)	Area under the curve (nM × min)	Lag time (min)
**Spearman's correlation coefficient (95% CI)**				
Overall	0.96 (0.95, 0.97)	-0.72 (-0.77, -0.66)	-0.55 (-0.61, -0.48)	0.80 (0.75, 0.84)
Rivaroxaban	0.96 (0.95, 0.98)	-0.73 (-0.79, -0.68)	-0.60 (-0.68, -0.51)	0.81 (0.77, 0.85)
Apixaban	0.94 (0.90, 0.98)	-0.60 (-0.69, -0.51)	-0.47 (-0.59, -0.36)	0.51 (0.40, 0.62)
Edoxaban	0.95 (0.90, 0.99)	-0.56 (-0.77, -0.34)	-0.25 (-0.59, -0.10)	0.75 (0.62, 0.88)
**Deming regression**				
**Slope (95% CI)**				
Overall	0.81 (0.73, 0.88)	-0.72 (-0.95, -0.56)	-15.89 (-19.04, -13.57)	0.01 (0.01, 0.02)
Rivaroxaban	0.81 (0.73, 0.90)	-0.73 (-0.98, -0.56)	-13.1 (-16.4, -10.8)	0.01 (0.01, 0.02)
Apixaban	1.04 (0.96, 1.11)	-1.02 (-1.49, -0.68)	-22.53 (-31.92, -17.26)	0.008 (0.005, 0.013)
Edoxaban	0.82 (0.80, 0.97)	-0.36 (-4.35, -0.22)	-14.51 (-248.98, 141.95)	0.003 (0.002, 0.008)
**Y-intercept (95% CI)**				
Overall	13.8 (7.1, 20.4)	313.9 (292.5, 339.1)	4842.3 (4607.2, 5154.9)	3.9 (3.6, 4.1)
Rivaroxaban	13.7 (5.7, 20.9)	307.7 (284.8, 336.5)	4560.0 (4313.9, 4866.8)	3.9 (3.6, 4.1)
Apixaban	-3.7 (-11.3, 4.0)	331.2 (288.0, 377.6)	5537.1 (4953.1, 6575.7)	3.85 (3.5, 4.2)
Edoxaban	14.7 (5.9, 22.8)	340.0 (306.0, 504.1)	4849.5 (-6309.3, 17808.2)	3.5 (3.1, 3.7)

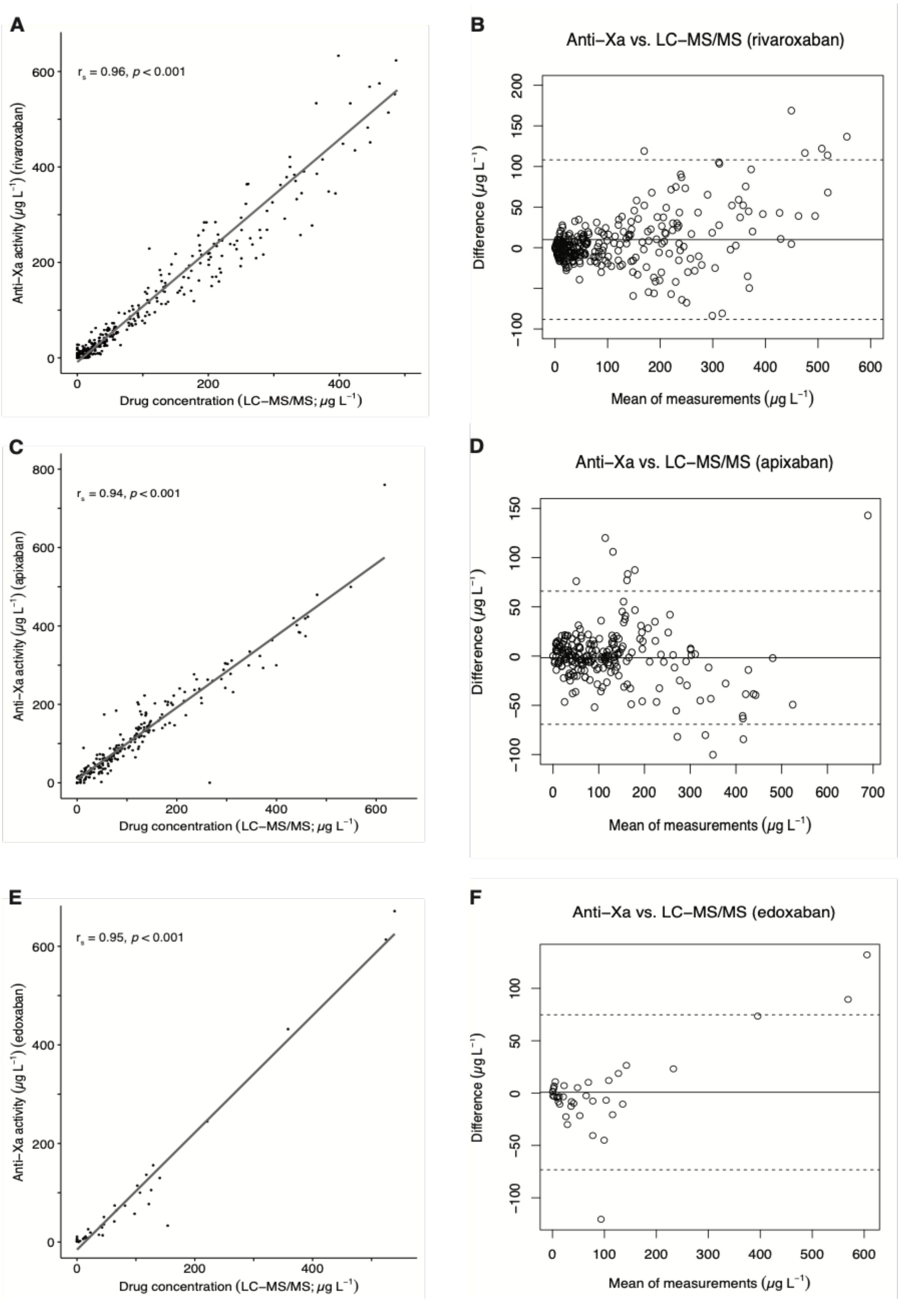

Figure S1. Association between anti-Xa measurements using the TECHNOCLONE assay and drug concentrations in 559 patients taking rivaroxaban, apixaban, or edoxaban in clinical practice. Scattergrams and Bland-Altman difference plots are given for rivaroxaban (A, B), apixaban (C, D), and edoxaban (E, F). Ultra-performance liquid chromatography-tandem mass spectrometry (LC-MS/MS) was used to determine drug levels. Spearman's correlation coefficient is given (r_s).
